# Effect of Nb Addition and Heat Input on Heat-Affected Zone Softening in High-Strength Low-Alloy Steel

**DOI:** 10.3390/ma15134503

**Published:** 2022-06-26

**Authors:** Feilong Wang, Gang Zhao, Yu Hou, Junpin Lin, Ba Li, Shujun Jia, Qingyou Liu, Gang Liu, Ping Yang

**Affiliations:** 1 State Key Laboratory of New Metal Materials, University of Science and Technology Beijing, Beijing 100083, China; wfeilong@126.com; 2 State Key Laboratory of Vanadium and Titanium Resources Comprehensive Utilization, Pangang Group Research Institute Co., Ltd., Panzhihua 617000, China; 3 Construction Project Management Branch of China National Petroleum Pipeline Network Group Co., Ltd., Langfang 065000, China; zhaogang1968@126.com (G.Z.); houyu@pipechina.com.cn (Y.H.); 4 Engineering Steel Research Institute, Central Iron and Steel Research Institute Company Limited, Beijing 100081, China; balicugb@sina.com (B.L.); jiashujun@cisri.com.cn (S.J.); liuqingyou@cisri.com.cn (Q.L.); 5 Beijing Advanced Innovation Center for Materials Genome Engineering, University of Science and Technology Beijing, Beijing 100083, China; westbrant@163.com; 6 School of Metallurgical Engineering, Anhui University of Technology, Maanshan 243032, China; y10190083@mail.ecust.edu.cn

**Keywords:** HAZ softening, welding thermal simulation, heat input, second phase precipitation, fine-grain heat-affected zone (FGHAZ)

## Abstract

The effect of both Nb content and heat input on the softening phenomenon of the heat-affected zone (HAZ) of low-alloy high-strength steel was studied through welding thermal simulation experiments. The microstructure evolution, density variation of geometrically necessary dislocation, microhardness distribution and the second phase precipitation behavior in HAZ was characterized and analyzed by combining the optical microscope, scanning electron microscope, high-resolution transmission electron microscope with microhardness tests. The results showed that the softening appeared in the fine-grain HAZ (FGHAZ) of the low-alloy high-strength steel with the polygonal ferrite and bainite microstructure. With an increase in Nb content, the FGHAZ softening was inhibited even with high heat input; however, the hardness shows little variation. On the one hand, the increase in the Nb content increased the volume fraction of high-strength bainite in the FGHAZ. On the other hand, the remarkable strengthening was produced by the equally distributed precipitation nanoparticles. As a result, the two factors were the main reason for the solution of the FGHAZ softening problem in the low-alloyed high-strength steel with the mixed microstructure of ferrite and bainite.

## 1. Introduction

Oil and gas energy are resources in nature that supports human survival and social progress [1]. However, there have been energy shortages in recent years [2]. On the one hand, the social development requires more energy; on the other hand, the mining coal and oil and gas on land is facing even greater challenges; for example, the oil and gas wells are depleted [3]. Therefore, more attentions concentrate on the exploitation of oil and gas resources in deep sea [4,5]. It was well worth noting that a large amount of pipeline steel is needed in both land and deep-sea mining, where the high-strength low-alloy (HSLA) steel was used due to the superior match between high strength and good low-temperature impact toughness [6,7].

The low-carbon HSLA steel is mainly used by welding, where the strength and heat input are the two main factors to affect the mechanical properties after welding [8]. In order to improve the yield strength of the steel, the thermo-mechanical control process (TMCP) is adopted without increasing the carbon equivalent, which ensures that sufficient weldability is attained [9]. It is noted that the higher the yield strength of base metals, the greater the possibility of the appearance of softening phenomenon in the weld heat-affected zone (HAZ). Additionally, the higher the heat input, the lower the hardness in the HAZ, leading to limited application [10]. Usually, the softening phenomenon potentially appeared in the coarse-grained zone, intercritical zone or fine-grained zone of HAZ, namely, CGHAZ, ICHAZ and FGHAZ, respectively [11,12,13]. Gu et al. [14] suggested that the larger-size polygonal ferrite (PF) and granular ferrite formed from the ununiform prior austenite resulted in softening phenomenon. In order to reduce the HAZ softening, researchers have put forward many methods [15,16]. Dong et al. [15] found that increasing the welding heat input could suppress the formation of martensite and reduce the microhardness of HAZ. However, there was relatively low welding heat input for obtaining the optimal mechanical properties. The low welding heat input and proper heat-treatment process are the effective way to inhibit the welding HAZ softening. Moreover, Li et al. [16] found that the addition of V element was the effective way to retard the softening phenomenon by refining the HAZ microstructure and promoting the nanoscale precipitation of the VC.

Moreover, the addition of Nb may be the more practicable way to solve the softening problems. Shanmugam et al. [17] designed the 700 MPa grade Nb/Ti-bearing HSLA steel without obvious HAZ softening phenomenon by precipitating the nano particles, as well as the similar microstructure constituents between the base metal and the welding HAZ. The effects of Nb addition in HSLA welding steel on the welding HAZ softening are also widely reported [18,19,20,21], by forming nano-precipitates and refining the microstructure. However, the study of welding softening problem rarely jointly focuses on the two factors, content of Nb addition and heat inputs, in HSLA steel manufactured by TMC. Given the above, the different contents of Nb were added to the HSLA steels, as well as the different heat inputs, to obtain the proper design concept of alloying elements and guidance of welding process parameters.

## 2. Materials and Methods

The Fe alloys were designed for studying the effect of Nb on welding HAZ to inhibit the softening phenomenon. The Fe alloys were smelted in a 50 kg high-frequency vacuum induction furnace, cast into steel ingots, and forged into a 60 mm × 80 mm ×100 mm billet, which are subjected to several pass rolling on the rolling mills after subjected to enough homogenizing treatment. The final thickness of the plates was about 30 mm, and the chemical composition of them is shown in Table 1, with an increase in Nb content from 0.062 wt.% to 0.112 wt.%.

Welding thermal simulation samples in dimensions of 61 mm × 10.5 mm × 10.5 mm were cut from the center of the thickness direction of the hot-rolled steel plate. For HSLA steels, there was low-temperature phase transformation below 500 °C, so the cooling time from 800 °C to 300 °C, namely t_8/3_, was used. At the same time, different welding heat inputs of 10, 25 and 35 kJ/cm are adopted, and the corresponding t_8/3_ times are 9.6, 24.95 and 33.6 s, respectively, according to the theoretical empirical formula proposed by D. Vwer [22]. The welding thermal simulation tests were carried out in the Gleeble-3800 thermal simulation test machine. Thermal histories of the different zones of HAZ were simulated and studied by setting a series of peak temperatures from 500 °C to 1300 °C. The heating rate to peak temperature was 100 °C/s, and the cooling rate from peak temperature to 800 °C was about 20 °C/s.

After mechanically grounding and polishing, specimens were examined by Olympus Lext 3100 optical microscope (OM) and FEI Quanta 650 field emission scanning electron microscope (SEM) for observing microstructure after etched in a mixed solution of 4% nitric acid and 96% absolute ethyl alcohol. The welding HAZ samples were studied at Zeiss Merlin SEM equipped with OXFORD NordlysNano electron backscatter diffractometer (EBSD) for obtaining the crystallographic data after they were electrolytic polished in a solution of 8% perchloric acid and 92% absolute ethyl alcohol. The carbon extraction replicas were used to observe the precipitation phases of the tested steel subjected to different welding thermal simulation process. After being removed by the 4% nital alcohol and cleaned in distilled water, the replicas were preserved on copper grids and observed in a high-resolution transmission electron microscope (HRTEM) Tecnai G^2^ F20. The INSTRON TUKON 2100 microhardness tester was used to measure the microhardness of the actual welded joint and the welded thermal simulation specimens, and the load was 200 g with the time of 10 s.

## 3. Results and Discussions

### 3.1. The Softening Phenomenon of Actual Welded Joint HAZ in HSLA Steel

**Figure 1:**
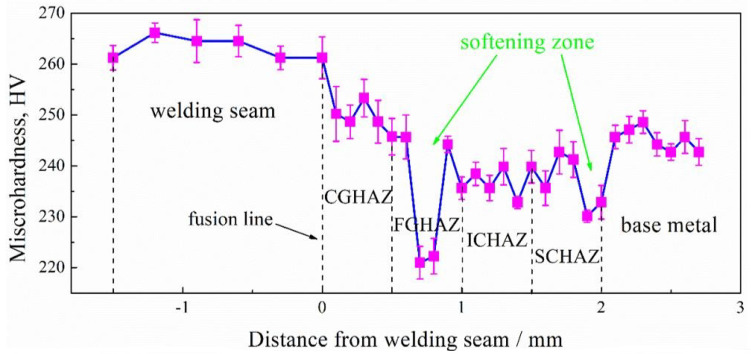
Schematic diagram of microhardness of actual welded joint.

Figure 1 shows the microhardness distribution of the 0Nb steel from the welding seam to the HAZ and then to the base metal on the actual welding joint. It can be seen that the hardness of welding seam was obviously higher than that of the base metal, as well as the hardness of CGHAZ slightly higher than that of the base metal. With an increase in the distance from the fusion line to base metal, the microhardness firstly decreased and then had a slight increase, especially in the FGHAZ, where the softening zone appeared (marked by arrow). From ICHAZ to base metal, there was no obvious softening phenomenon except the slight decrease in hardness at the binding zone between subcritical zone (SCHAZ) and base metal.

**Figure 2:**
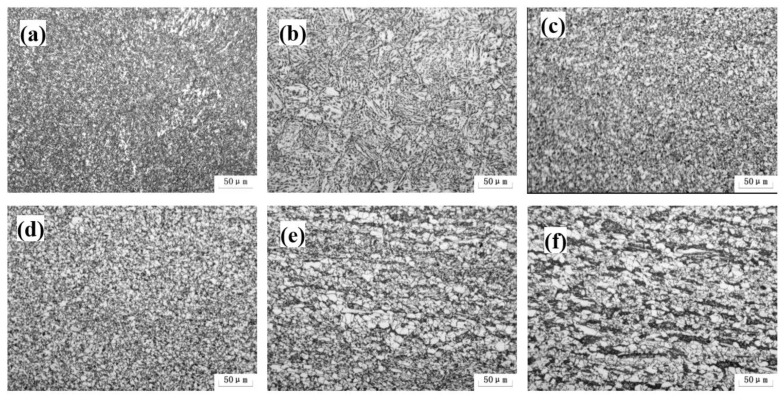
Microstructure of actual welded joints of 0 Nb steel: (**a**) weld seaming; (**b**) CGHAZ; (**c**) FGHAZ; (**d**) ICHAZ; (**e**) SCHAZ; (**f**) base metal.

The actual welded joint microstructure of 0Nb steel is displayed in Figure 2. The typical acicular ferrite (AF) microstructure was observed in welding seam. The welding HAZ from CGHAZ, FGHAZ, IGHAZ and SCHAZ were exhibited from Figure 2b–e, respectively, where the microstructure varied from the AF to the mixed PF, AF and pearlite (P). The microstructure of the welding HAZ is PF, bainite (B) and Perlite (P), which were attributed to the different peak temperatures and cooling rates [23]. The higher the temperature, the faster the cooling rate. So, the non-equilibrium state was introduced that induced the transformation of undercooled austenite into metastable microstructure such as AF and B.

**Figure 3:**
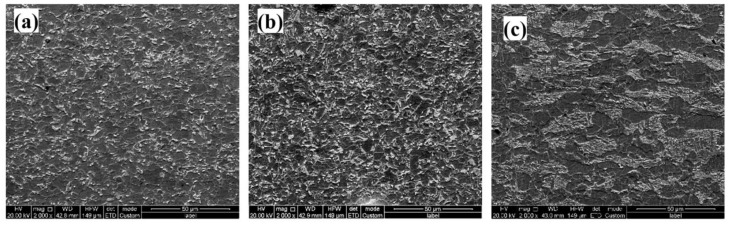
Microstructure of actual welded joints of 0 Nb steel: (**a**) FGHAZ; (**b**) SCHAZ; (**c**) base metal.

Further characterized by SEM, the high magnification microstructure images of the FGHAZ, SCHAZ and based metal were revealed in Figure 3. The base metal possessed PF, B and P mixed microstructure, which was consistent with the OM observation (Figure 2f and Figure 3c). The P was spheroidized because the lath P was not found. It can be seen that plenty of M/A constituents were distributed in FGHAZ and SCHAZ. The content and mean size of M/A constituents in FGHAZ were lower and smaller than that of SCHAZ. It can be seen that the bainite structure in the SCHAZ has undergone a certain degree of recovery decomposition (Figure 3b).

The ICHAZ has undergone partial austenitization, which is still transformed into bainite after cooling (Figure 2d). In contrast, the microstructure in the FGHAZ was subjected to complete austenitization, which has relatively uniform distribution of elements. The elements, such as C, Mn, Ni and Cu, that enlarge the austenite region diffused into the reversed austenite [24,25], which transformed into plenty of the hard-phase B. This was because these elements decreased the transformation temperature, and the high hardenability and austenite undercooling degree proceeded the metastable transformation of B. When the partial reversed austenite transformation occurred, the higher contents of above elements in reversed austenite tended to transform into hard M/A constituents.

**Figure 4:**
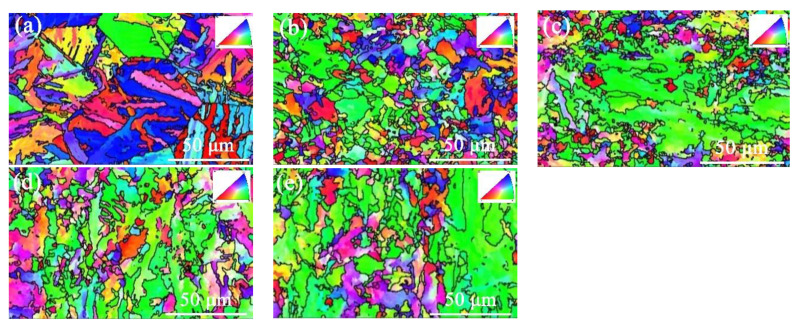
IPF images of actual welded joints: (**a**) CGHAZ; (**b**) FGHAZ; (**c**) ICHAZ; (**d**) SCHAZ; (**e**) base metal.

Figure 4 shows the inverse pole figures (IPF) of different HAZs of the actual welding actual welded joints. The black lines are the high-angle grain boundaries (HAGB, above 15°). The CGHAZ mainly contained bainite ferrite with low-density HAGBs (Figure 4a). The prior austenite grain boundary could be clearly observed, and it was divided by ferrite laths or lath bundles. The lath boundaries and the bundle boundaries were both HAGBs. Adjacent to the CGHAZ, it was the FGHAZ, which were composed of a large amount of fine-grained ferrite, leading to the refined microstructure about 20%, as shown in Figure 4b.

Figure 4c shows the ICHAZ near the FGHAZ, more fine-grained ferrite is formed at the prior austenite grain boundary after rapid cooling due to the partial austenitization, resulting in the unevenly distributed microstructure. In contrast, the microstructure in SCHAZ and the base metal had the similar morphology, and both of them contained bainite and ferrite (Figure 4d,f). It was noted that the fine P was not detected because the size of Fe_3_C was smaller the EBSD scanning step size.

The line intercept method was used to calculate the effective grain sizes, and the results are listed in Table 2. Compared with the base metal, after thermal cycles at different temperatures, the microstructure of each area in HAZ has been refined to a certain extent. Furthermore, the microstructure in FGHAZ was the finest, but the degree of grain refinement was small. Therefore, the enhancement of the strength of the refined region by the refined grain is limited.

**Figure 5:**
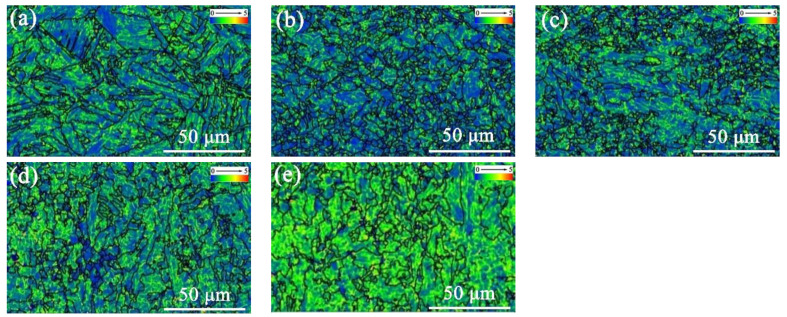
Local misorientation maps of actual welded joints: (**a**) CGHAZ; (**b**) FGHAZ; (**c**) ICHAZ; (**d**) SCHAZ; (**e**) base metal.

The local misorientation function of EBSD was further used to characterize the local difference in each area in HAZ, combining with the HAGBs in black lines (Figure 5). This function of EBSD depicted the variation in the density and distribution of misorientation less than 5° inside the grain through the color change. The color changing from blue to red implied that the content of misorientation less than 5° inside the grain changes from more to less. The higher the content of the low-angle misorientation, the more uniform the stress distribution inside the grains. The local misorientation distribution maps displayed the degree of local stress concentration, and can also be used to indirectly characterize the change in dislocation density [26]. The relationship between the geometric dislocation density and the local misorientation difference in the crystal is given by Formula (1):

(1) ρ=2θ/μb

where *ρ* is the geometric dislocation density, *θ* is the average value of the local orientation difference, *μ* is the scan step size, and *b* is the Burgers vector. It can be seen from Formula (1) that the geometric dislocation density in the crystal increases with the increase in the local misorientation difference.

In Figure 5, the color changing from blue to green and then to orange indicated that the degree of stress concentration in matrix was increasing, showing the increase in dislocation density. Compared with the base material, the dislocation density of the microstructure in each region of the HAZ is lower, but it can be seen from the color contrast that the dislocation density in FGHAZ is lowest.

**Figure 6:**
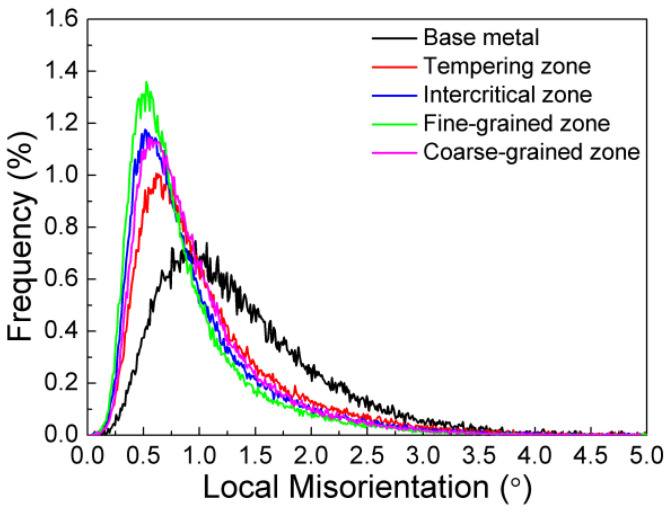
Quantitative analysis of local misorientation of various peak temperatures.

In order to quantitatively compare the dislocation density of various microstructures, Figure 6 shows the distribution of local misorientation. It can be clearly compared that the dislocation density in FGHAZ is the lowest. In addition, Formula (2) gives the relationship between dislocation density and strength of metallic materials [27]:

(2) $$\sigma =\sigma_{0}+{2\alpha Gb\rho }^{\frac{1}{2}}$$

where *σ*_0_ is the initial strength of the material, α is the lattice constant, *G* is the shear modulus, *b* is the Burgers vector, and *ρ* is the dislocation density. Formula (2) shows that the strength of the material is proportional to the dislocation density, and correspondingly, the hardness is also proportional to the dislocation density. Based on the above analysis, it can be seen that the decrease in dislocation density is an important reason that induces the HAZ softening appearance in the welding HSLA steels.

### 3.2. Effect of Simulated Heat Input on the HAZ Softening in Low-Alloyed High-Strength Steel

**Figure 7:**
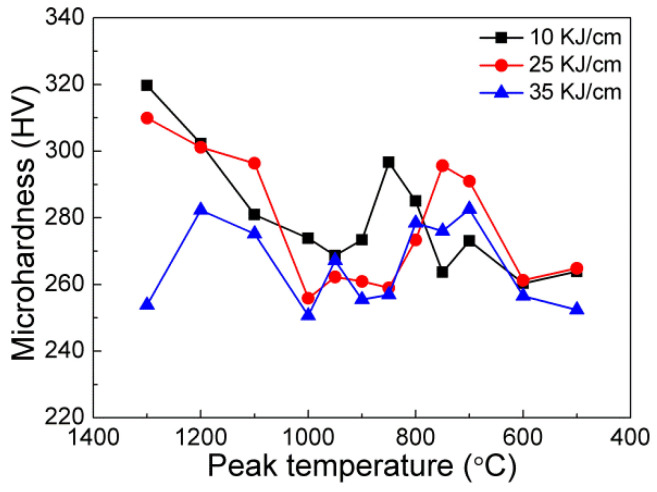

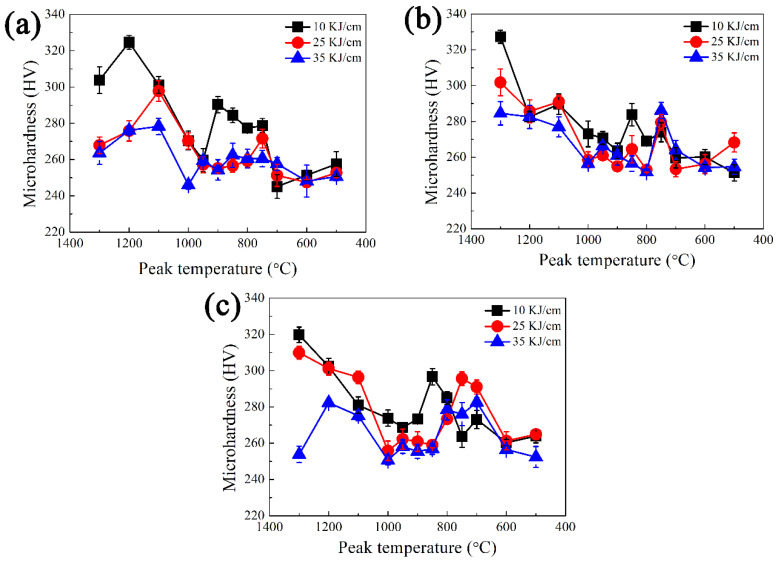
Effect of heat input on microhardness subjected to various peak temperatures and different Nb contents: (**a**) 1#−0.062% Nb; (**b**) 2#−0.089% Nb; (**c**) 3#−0.112% Nb.

Figure 7 shows the relationship between the microhardness and the peak temperature of the three Nb-bearing steels under different welding heat input conditions. There was the biggest microhardness difference when the heat input is 10 kJ/cm for Nb-bearing steel, and the microhardness difference decreased as the heat input increased. It was worth noting that the lower the heat input, the higher the hardness of the softening area. Therefore, the low heat input can improve the welding softening problem. Moreover, with an addition of the Nb content, the microhardness in softening area reached the same level as the hardness of the base metal (about 245 HV), or even higher. As the Nb content increased, the overall fluctuation of microhardness decreased, and the average hardness level increased, compared with that of 0Nb steel. There, the FGHAZ softening phenomenon of the HSLA steel can be reduced by combining the low welding heat input and the proper addition of Nb.

**Figure 8:**
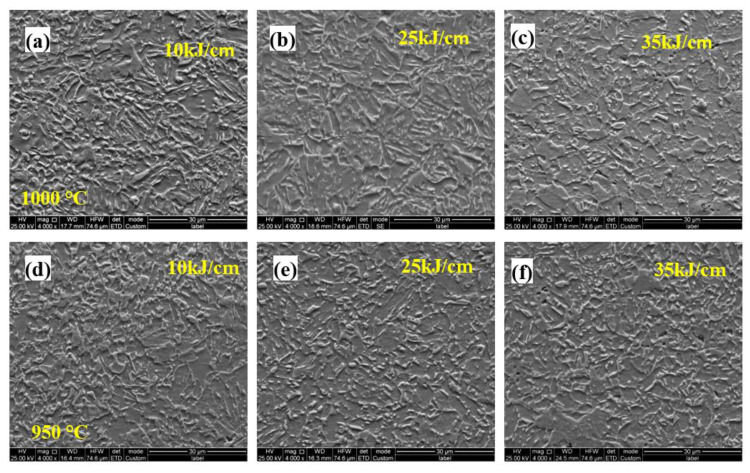
Microstructure of FGHAZ with 0.062% Nb content under various heat input and peak temperature: (**a**) 10 kJ/cm at 1000 °C; (**b**) 25 kJ/cm at 1000 °C; (**c**) 30 kJ/cm at 1000 °C; (**d**) 10 kJ/cm at 950 °C; (**e**) 25 kJ/cm at 950 °C; (**f**) 30 kJ/cm at 950 °C.

Figure 8 shows the microstructure evolution of the 0.062% Nb steel subjected to the different heat inputs and peak temperatures in FGHAZ. The microstructure was mainly PF, granular B (GB), and bainite ferrite. With an increase in heat input, the microstructure coarsened. Additionally, there was no obvious microstructure evolution when the peak temperature increased from 950 °C to 1000 °C.

**Figure 9:**
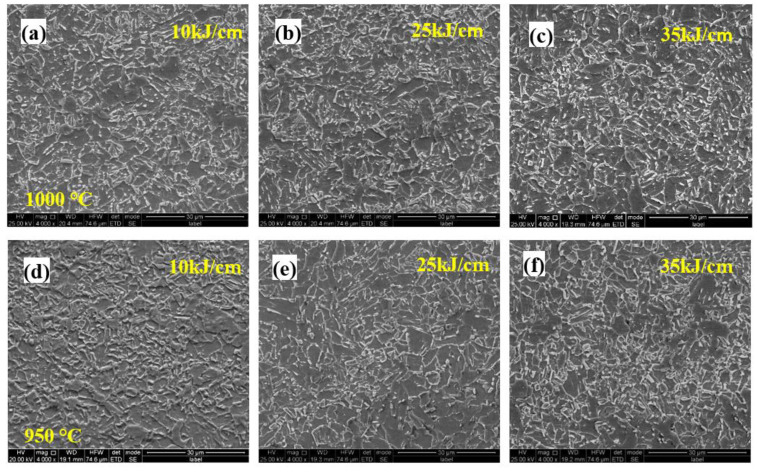
Microstructure of FGHAZ with 0.089% Nb content under various heat input and peak temperature: (**a**) 10 kJ/cm at 1000 °C; (**b**) 25 kJ/cm at 1000 °C; (**c**) 30 kJ/cm at 1000 °C; (**d**) 10 kJ/cm at 950 °C; (**e**) 25 kJ/cm at 950 °C; (**f**) 30 kJ/cm at 950 °C.

Figure 9 displays the microstructure evolution of the 0.089% Nb steel subjected to the different heat inputs and peak temperatures in FGHAZ. Compared with 0.062% Nb steel, more M/A constituents with larger size appeared, and the content of PF decreased. With an increase in heat input, there was no obvious microstructure coarsening, but the portion of M/A constituents increased, potentially showing the increasing hardness. Additionally, the microstructure evolution is little when the peak temperature increased from 950 °C to 1000 °C.

**Figure 10:**
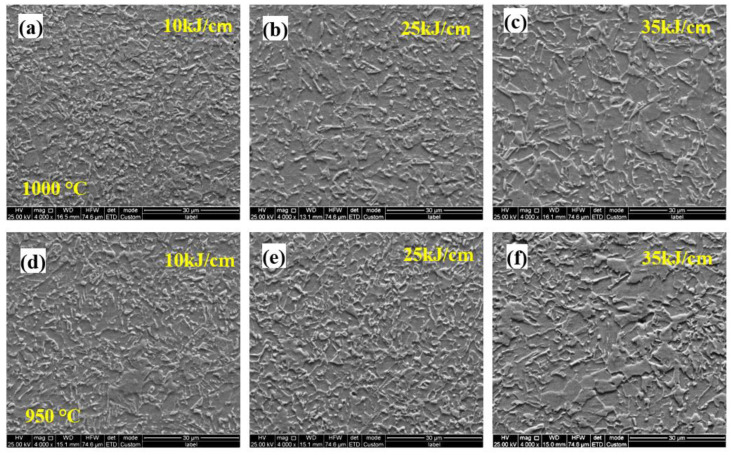
Microstructure of FGHAZ with 0.112Nb content under various heat input and peak temperature: (**a**) 10 kJ/cm at 1000 °C; (**b**) 25 kJ/cm at 1000 °C; (**c**) 30 kJ/cm at 1000 °C; (**d**) 10 kJ/cm at 950 °C; (**e**) 25 kJ/cm at 950 °C; (**f**) 30 kJ/cm at 950 °C.

Figure 10 depicts the microstructure evolution of the 0.112% Nb steel subjected to the different heat inputs and peak temperatures in FGHAZ. Compared with 0.089% Nb steel, the content of M/A constituents decreased, while the content of PF increased. The microstructure types and constituents were similar with that of 0.062% Nb steel. With an increase in heat input, the microstructure coarsened, and the counts of M/A constituents decreased, implying the microhardness decreased than that of the 0.089% Nb steel, which was consistent with the results of microhardness of different peak temperatures (Figure 7). As the peak temperature increased from the 950 °C to 1000 °C in FGHAZ, there was no obvious microstructure evolution, indicating that the peak temperature had little effect on decreasing the hardness in the same HAZs. Furthermore, decreasing the welding heat input is beneficial to improve the softening phenomenon of the FGHAZ. However, even under the highest heat input conditions, there is no softening in the FGHAZ, indicating that heat input is not the main reason for the reduction in softening.

### 3.3. Effect of Nb Microalloying on the HAZ Softening in HSLA Steel

**Figure 11:**
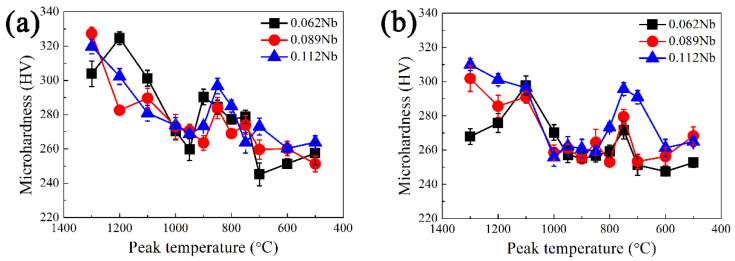

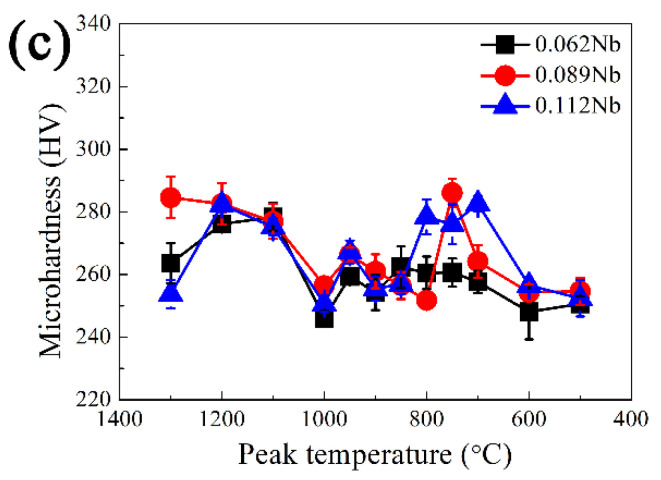
Microhardness of test steels under different Nb contents with (**a**) 10 kJ/cm, (**b**) 25 kJ/cm and (**c**) 30 kJ/cm heat inputs.

Figure 11 shows the microhardness variation of different Nb-bearing steels with welding heat input conditions, potentially indicating the relationship between the microhardness and the peak temperatures and Nb contents. The trend of microhardness decreased as the peak temperature decreased, which strongly depended on the microstructure evolution. The microhardness of different HAZs except the SCHAZ had relatively little variation among the Nb-bearing HSLA steels. The results suggested that the increased Nb content could improve the HAZ softening phenomenon of the HSLA steel. However, it needs to be clarified that there was no obvious microhardness variation with the Nb content variation, indicating the effect of Nb microalloying on improving the softening phenomenon of the HSLA steel is limited. The microhardness in FGHAZ subjected is all higher than that of base metal regardless of the niobium content, indicating that the addition of Nb can significantly suppress the welding HAZ softening (Figure 11a–c).

As the Nb content increases, the phase transformation temperature decreases. The HSLA steels was fully austenitized within the temperature range of the FGHAZ, and more B microstructure is still obtained after cooling. Nb increases the hardenability of austenite, and if in solid solution, more B is transformed instead of F during cooling. Therefore, the hardness is not reduced, and no softening phenomenon occurs. Moreover, the Nb element was still the important factor to influence the microhardness. It was because the Nb atoms in solution state could increase the hardenability, which prompted the hard phase transformation. On the other hand, the Nb precipitation particles also could provide a considerable precipitation strengthening increment, which inhibited the softening phenomenon.

**Figure 12:**
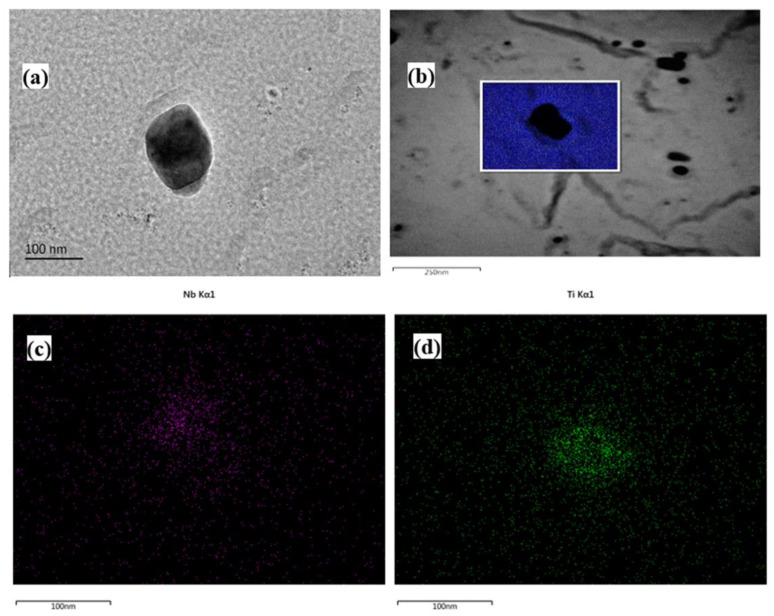
Morphology and surface scan of large size precipitation of experiment steel at peak temperature of 950 °C under the heat input of 10 kJ/cm: (**a**) precipitation particle morphology; (**b**–**d**) the particle mapping and element distribution.

**Figure 13:**
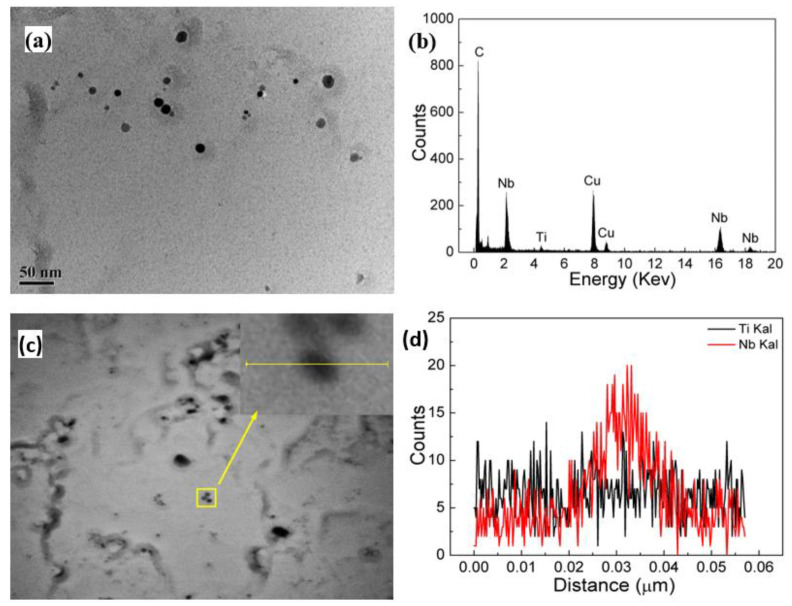
Precipitation morphology and chemical composition of experiment steel at peak temperature of 950 °C under the heat input of 10 kJ/cm: (**a**) precipitation morphology; (**b**) point scanning result; (**c**) line scanning location; (**d**) line scanning result.

**Figure 14:**
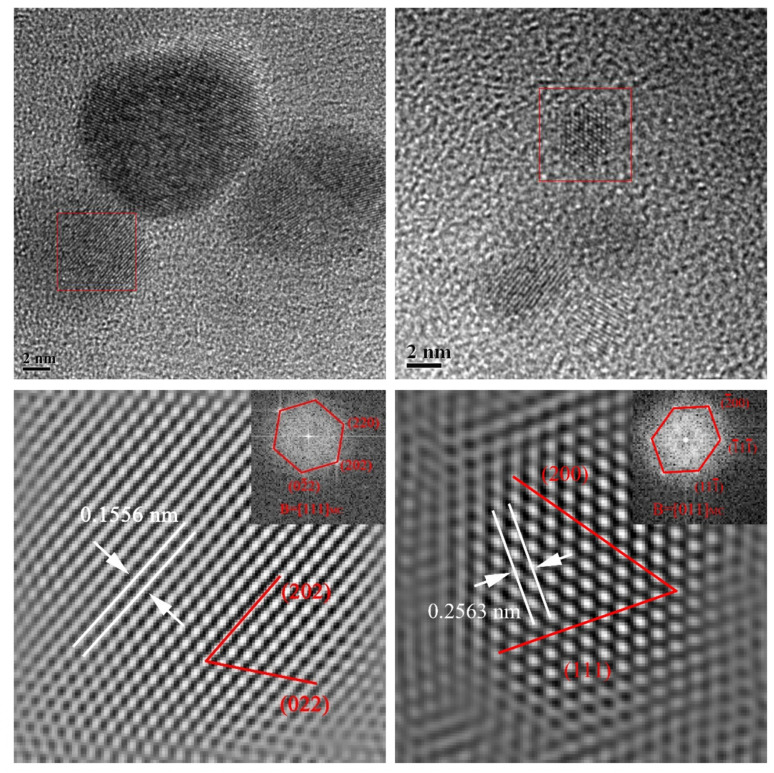
High resolution TEM images of nanometer precipitation at peak temperature of 950 °C under the heat input of 10 kJ/cm.

As shown in Figure 12, Figure 13 and Figure 14, the precipitation particles in different scale range are characterized by TEM. The large-size precipitation particle was near nearly spherical, and the upper part of the selected particle was mainly Nb-rich area and the lower part is Ti-rich area. The EDS mapping results showed that the Ti content in the particle was higher that Nb content, and tended to concentrate in center part, which may be because TiC or TiN firstly precipitated at high temperature, and Nb gradually diffused into the precipitated particle as the temperature decreased [28]. Usually, the higher the precipitation temperature, the larger size the precipitation particles. Many particles about 10~50 nm were observed in Figure 13 basing on the carbon film extraction method, where the replicas were stored in a Cu grid. The EDS result (point and line) shows that the content of Nb was higher than the content of Ti, and the ratio of Nb/Ti is higher than the mean chemical composition design, implying the precipitation particles below 50 nm were precipitated in lower temperature with more Nb content.

Further characterized by the high-resolution TEM, the nano-precipitation particles below 10 nm were mainly spherical. It was found that the particles were along the [011] and [111] zone axis. The precipitation particle size is too small to be detected for the chemical composition, especially Nb, but the images show that the interplanar crystal spacing was between the theoretical values of NbC and TiC, where the measured interplanar crystal spacing was 0.1556 nm for {200}_MC_ and 0.2563 nm for {220}_MC_, indicating that these particles were the Nb/Ti jointly precipitation particles, namely, (Nb, Ti)C [24]. It was worth noting that the round particles precipitated in the single austenite phase region and obeyed the cube-on-cube parallel orientation relationship, resulting in the spherical particle morphology [29].

**Figure 15:**
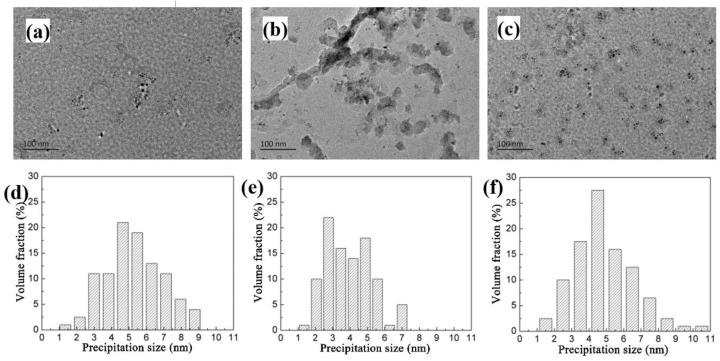
Precipitation and quantitative statistics of the (Ti, Nb)C under different Nb contents: (**a**) 06 Nb, (**b**) 09 Nb and (**c**) 11 Nb of precipitation morphology and distribution; (**d**–**f**) quantitative statistics of 06 Nb, 09 Nb, and 11 Nb, respectively.

This nano-scale precipitated phase has a precipitation strengthening effect and can effectively improve the strength and hardness of the matrix. The second phase precipitation distribution and quantitative statistical results of the three experimental steels are shown in Figure 15, where the peak temperature and heat input are 950 °C and 10 kJ/cm, respectively. As the Nb content increases, the mean round precipitation particle size was about 4.69 nm, 4.91 nm and 5.29 nm, and the number of precipitation particles is 77, 91 and 215, respectively (Figure 15a–c). Additionally, the quantitative statistics shows the particle size distribution is Gaussian distribution. According to the Orowan strengthening mechanism between the dislocation movement, and the precipitation particle size, the precipitation strengthening increment can be described as following [30]:

(3) $$YS_{P}=8995\times \frac{f^{0.5}}{d}\ln\left(2.417d\right)$$

where the *f* is the precipitation phase volume fraction, and *d* is the particle size, nm. The strengthening of (Ti, Nb)C increases when the Nb content increases, because the particle size just reaches the cut-through mechanism and the bypass mechanism. Moreover, the solid-solution Nb atoms increase with the Nb addition, which increases the hardenability. The hardenability promotes the metastable microstructure transformation, which increases the dislocation density, and this is also a considerable strengthening method.

In summary, the precipitation of the nano-scale second phase is another crucial reason for Nb microalloying to improve HAZ softening.

## 4. Conclusions

Different contents of the Nb element were added to the pipeline steel to study the effect on the HAZ softening phenomenon, as well as the introduction of different welding heat inputs. According to the characterization by OM, SEM, EBSD and TEM, and analysis, the main results are as follows:

(1) The microstructure varied from the AF to the mixed PF, AF and P from the welding seam to the base metal. The further the distance from the welding seam, the closer the equilibrium-state microstructure was obtained, such as PF and P, which potentially caused the softening phenomenon in FGHAZ.
(2) Decreasing the welding heat input is beneficial to improve the softening phenomenon of the FGHAZ. However, even under the highest heat input conditions, there is no softening in the FGHAZ, indicating that heat input is not the main reason for the softening reduce.
(3) The increased Nb addition effectively suppresses the FGHAZ softening phenomenon; however, it is limited when the content of Nb reaches more than 0.1%. On the one hand, the increasing Nb content increases the content of hard-phase B; on the other hand, the precipitation of the nano-scale second phase particles, (Ti, Nb)C, plays a crucial role in precipitation strengthening.

## Figures and Tables

**Table 1:** Chemical compositions of experimental steels (wt.%).

Samples	C	Si	Mn	P	S	Nb	Ti	Mo	Ni	Cr	Cu
0 Nb	0.051	0.21	1.81	0.0050	0.0033	/	0.012	0.254	0.22	0.206	0.21
06 Nb	0.051	0.19	1.79	0.0048	0.0034	0.062	0.013	0.255	0.21	0.207	0.21
09 Nb	0.052	0.18	1.82	0.0058	0.0042	0.089	0.012	0.253	0.21	0.206	0.21
11 Nb	0.048	0.20	1.78	0.0053	0.0028	0.112	0.010	0.254	0.22	0.209	0.21

**Table 2:** Effective grain size of different HAZs measured by line intercept method of EBSD.

HAZ and Base Metal	CGHAZ	FGHAZ	ICHAZ	SCHAZ	Base Metal
RD (μm)	4.40	3.34	3.45	3.80	4.45
ND (μm)	3.95	3.24	3.27	4.30	5.50
